# Complement activating antibodies to myelin oligodendrocyte glycoprotein in neuromyelitis optica and related disorders

**DOI:** 10.1186/1742-2094-8-184

**Published:** 2011-12-28

**Authors:** Simone Mader, Viktoria Gredler, Kathrin Schanda, Kevin Rostasy, Irena Dujmovic, Kristian Pfaller, Andreas Lutterotti, Sven Jarius, Franziska Di Pauli, Bettina Kuenz, Rainer Ehling, Harald Hegen, Florian Deisenhammer, Fahmy Aboul-Enein, Maria K Storch, Peter Koson, Jelena Drulovic, Wolfgang Kristoferitsch, Thomas Berger, Markus Reindl

**Affiliations:** 1 Clinical Department of Neurology, Innsbruck Medical University, Innsbruck, Austria; 2 Department of Pediatrics IV, Division of Pediatric Neurology and Inborn Errors of Metabolism, Innsbruck Medical University, Innsbruck, Austria; 3 Clinic of Neurology, Clinical Center of Serbia, Belgrade, Serbia; 4 Division of Histology and Embryology, Innsbruck Medical University, Austria; 5 Division of Molecular Neuroimmunology, Department of Neurology, University of Heidelberg, Heidelberg, Germany; 6 Department of Neurology, SMZ-Ost Donauspital, Vienna, Austria; 7 Department of Neurology, Medical University of Graz, Graz, Austria; 8 Department of Neurology, Slovak Medical University, University Hospital Ruzinov, Bratislava, Slovakia; 9 Institute of Neuroimmunology, Slovak Academy of Sciences, Bratislava, Slovakia; 10 Faculty of Medicine, University of Belgrade, Belgrade, Serbia; 11 Karl Landsteiner Institute for Neuroimmunological and Neurodegenerative Disorders, Vienna, Austria

**Keywords:** Neuromyelitis optica, autoantibodies, myelin oligodendrocyte glycoprotein, aquaporin-4, complement mediated cytotoxicity, biomarker

## Abstract

**Background:**

Serum autoantibodies against the water channel aquaporin-4 (AQP4) are important diagnostic biomarkers and pathogenic factors for neuromyelitis optica (NMO). However, AQP4-IgG are absent in 5-40% of all NMO patients and the target of the autoimmune response in these patients is unknown. Since recent studies indicate that autoimmune responses to myelin oligodendrocyte glycoprotein (MOG) can induce an NMO-like disease in experimental animal models, we speculate that MOG might be an autoantigen in AQP4-IgG seronegative NMO. Although high-titer autoantibodies to human native MOG were mainly detected in a subgroup of pediatric acute disseminated encephalomyelitis (ADEM) and multiple sclerosis (MS) patients, their role in NMO and High-risk NMO (HR-NMO; recurrent optic neuritis-rON or longitudinally extensive transverse myelitis-LETM) remains unresolved.

**Results:**

We analyzed patients with definite NMO (n = 45), HR-NMO (n = 53), ADEM (n = 33), clinically isolated syndromes presenting with myelitis or optic neuritis (CIS, n = 32), MS (n = 71) and controls (n = 101; 24 other neurological diseases-OND, 27 systemic lupus erythematosus-SLE and 50 healthy subjects) for serum IgG to MOG and AQP4. Furthermore, we investigated whether these antibodies can mediate complement dependent cytotoxicity (CDC). AQP4-IgG was found in patients with NMO (n = 43, 96%), HR-NMO (n = 32, 60%) and in one CIS patient (3%), but was absent in ADEM, MS and controls. High-titer MOG-IgG was found in patients with ADEM (n = 14, 42%), NMO (n = 3, 7%), HR-NMO (n = 7, 13%, 5 rON and 2 LETM), CIS (n = 2, 6%), MS (n = 2, 3%) and controls (n = 3, 3%, two SLE and one OND). Two of the three MOG-IgG positive NMO patients and all seven MOG-IgG positive HR-NMO patients were negative for AQP4-IgG. Thus, MOG-IgG were found in both AQP4-IgG seronegative NMO patients and seven of 21 (33%) AQP4-IgG negative HR-NMO patients. Antibodies to MOG and AQP4 were predominantly of the IgG1 subtype, and were able to mediate CDC at high-titer levels.

**Conclusions:**

We could show for the first time that a subset of AQP4-IgG seronegative patients with NMO and HR-NMO exhibit a MOG-IgG mediated immune response, whereas MOG is not a target antigen in cases with an AQP4-directed humoral immune response.

## Background

Neuromyelitis optica (NMO), a severe inflammatory demyelinating disorder, has gained increasing interest since the discovery of serum NMO-IgG autoantibodies targeting the aquaporin-4 (AQP4) water channel protein [1,2]. The detection of this highly specific biomarker resulted in the incorporation of the NMO-IgG serostatus in the diagnostic criteria of NMO [3]. An early differentiation from multiple sclerosis (MS) is highly important, due to differences in prognosis and therapy of NMO patients. The target antigen AQP4 is localized on astrocytic endfeet [4] and is expressed as full length M1 or shorter M23 AQP4 isoform [5,6]. Recently, serum anti-AQP4 antibodies were shown to bind primarily to the shorter M23 AQP4 isoform [7-9], which is of high diagnostic relevance due to an increased sensitivity of NMO-IgG analysis. Antibodies to AQP4 are also frequently detected in so called "High-risk NMO" (HR-NMO) patients not fulfilling all diagnostic criteria for NMO, who present with NMO-associated symptoms like recurrent optic neuritis (ON) or longitudinally extensive transverse myelitis (LETM) extending more than three vertebral segments [10]. NMO-IgG seropositivity was shown to be predictive for a poor visual outcome and the development of NMO in patients with recurrent ON [11,12]. Furthermore, the detection of AQP4-IgG in patients with a first episode of LETM extending ≥ three vertebral segments was associated with further relapses of LETM or ON, in some cases even within half a year [13]. Therefore, NMO and HR-NMO patients (recurrent ON or monophasic/recurrent LETM) are also classified as NMO-spectrum disorders (NMOSD) [10]. However, AQP4-IgG are missing in 5-40% of these patients, depending on the immunoassay used [9,12,14-16]. It is not yet known whether autoantibodies to other central nervous system (CNS) specific antigens are present in patients with NMO and HR-NMO [17].

Recent experimental studies indicated that myelin oligodendrocyte glycoprotein (MOG), a glycoprotein localized on the outer surface of the myelin sheath and oligodendrocytes [18], might be a target antigen in NMO. Two *in vivo *studies demonstrated spontaneous development of NMO-like symptoms with severe opticospinal experimental autoimmune encephalomyelitis (EAE) in a double-transgenic opticospinal EAE (OSE) mouse model expressing T cell and B cell receptors specific for MOG [19,20]. This mouse strain closely resembles human NMO by exhibiting prototypical inflammatory demyelinating lesions in the optic nerve and spinal cord. Furthermore, the animals were found to exhibit highly positive serum MOG-IgG1 antibodies [19]. Additionally, there are several reports demonstrating the induction of an NMO-like disease following immunization of certain rat strains with MOG [21-23].

Whereas in humans anti-MOG antibodies in MS have been extensively investigated, their role in NMO has not been adressed so far. High-titer IgG autoantibodies to conformational epitopes of MOG (MOG-IgG) were detected in a subgroup of pediatric patients with acute disseminated encephalomyelitis (ADEM) and MS, but rarely in adult-onset MS [24-29]. A possible role of MOG-IgG antibodies in NMO-related diseases is supported by recent findings of our group, demonstrating an increased frequency of MOG-IgG in pediatric patients with recurrent ON compared to monophasic ON subjects (Rostasy K, Mader S, Schanda K, Huppke P, Gärtner J, Kraus V, Karenfort M, Tibussek D, Blaschek A, Kornek B, Leitz S, Schimmel M, Di Pauli F, Berger T, Reindl M: Anti-MOG antibodies in children with optic neuritis, in press). However, so far only one study using the bacterially expressed extracellular domain of MOG as antigen described the occurrence of a humoral immune response to MOG in four NMO patients [30].

Therefore, we decided to investigate the frequency and titer levels of IgG antibodies to MOG and AQP4 in a multicenter study of patients with CNS demyelinating diseases using a live cell staining immunofluorescence assay with HEK-293A cells transfected with either AQP4 or MOG [9,24]. In addition, we analyzed the IgG subtypes of antibodies directed to MOG and AQP4 and their ability to activate the complement cascade in a subset of patients.

## Results

### Serum AQP4-IgG and high-titer MOG-IgG antibodies in different disease groups

**Figure 1:**
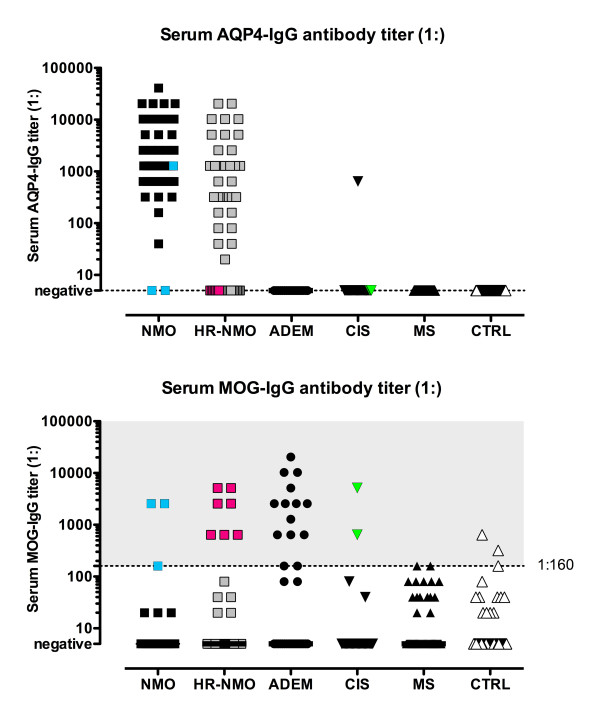
**Serum MOG-IgG and AQP4-IgG antibody titer levels in CNS demyelinating diseases and controls**. Serum AQP4-IgG (upper panel) were exclusively detected in serum samples of patients with NMO and HR-NMO and one CIS patient, but not in any subjects with ADEM, MS and CTRL. Serum MOG-IgG (lower panel) were present at highest titers (≥ 1:160, indicated by a dotted vertical line) in a cohort of patients with ADEM, AQP4-IgG seronegative HR-NMO (pink squares) and in both AQP4-IgG seronegative NMO subjects (blue squares, both at a MOG-IgG titer of 1:2,560) as well as in two CIS samples (green triangles). One patient (NMO) was found to be double positive for MOG-IgG (blue square at the threshold level of 1:160, lower panel) and AQP4-IgG (blue square at a titer of 1:1,280; upper panel). Coloured icons indicate individual patients in the upper and lower panel.

Using our assay with M23 AQP4 transfected HEK-293A cells, we detected significantly increased frequencies of serum AQP4-IgG in NMO (n = 43, 96%) and HR-NMO (n = 32, 60%; Table 1). Median AQP4-IgG titers of seropositive patients were 1:1,280 (1:40-1:40,960) in NMO and 1:1,280 (1:20-1:20,480) in HR-NMO (Figure 1). In addition, AQP4-IgG (titer 1:640) was detected in one patient with clinically isolated syndrome (CIS) presenting with myelitis. AQP4-IgG antibodies were absent in two patients with NMO (4%), 21 patients with HR-NMO (40%), 31 CIS patients (97%) and all patients with ADEM and MS as well as all controls (CTRL) including patients with systemic lupus erythematosus (SLE), other neurological diseases (OND) and healthy individuals (Table 1).

**Table 1:** Serum IgG antibodies directed to AQP4 and MOG (high-titer MOG-IgG antibodies at ≥ 1:160) in patients with CNS demyelinating diseases

Disease	Number	Females^1^	Age (y)^2^	AQP4-IgG^1,3^	MOG-IgG^1,3^
NMO	45	41 (91%) *	50 (2-80)	43 (96%) §	3 (7%)
				1,280	2,560
				(40-40,960)	(160-2,560)

HR-NMO	53	34 (64%)	48 (13-74)	32 (60%) §	7 (13%)
				1,280	2,560
				(20-20,480)	(640-5,120)

ADEM	33	20 (61%)	12 (2-52) §	0 (0%)	14 (42%) §
				-	2,560
					(160-20,480)

CIS	32	22 (69%)	34 (5-57) +	1 (3%)	2 (6%)
				640	(640; 5,120)

MS	71	43 (61%)	40 (14-66)	0 (0%)	2 (3%)
					(160; 160)

CTRL	101	79 (78%)	43 (14-84)	0 (0%)	3 (3%)
					320
					(160-640)

p-value		0.003	< 0.001	< 0.001	< 0.001

P-value: groups were compared using^1 ^Chi-Square test with Fisher's exact post-hoc test and^2 ^Kruskal-Wallis test with Dunn's post-hoc test (data are shown as median with range);^3 ^median titer level (range) of MOG-IgG or AQP4-IgG seropositive samples; * statistically different from HR-NMO, ADEM and MS; + statistically significant from NMO, HR-NMO and ADEM; § statistically significant from all other groups (p-values were corrected for multiple comparisons). Abbreviations: y = years, NMO = Neuromyelitis optica, HR-NMO = High-risk NMO, ADEM = acute disseminated encephalomyelitis, CIS = clinically isolated syndrome, MS = multiple sclerosis, CTRL = controls.

In addition to AQP4-IgG, we analyzed antibodies directed to natively folded human MOG expressed on the surface of human cells in the same set of patients (Table 1 and Figure 1). The frequency of high-titer (≥ 1:160) serum MOG-IgG antibodies was significantly increased in patients with ADEM (n = 14, 42%). However, high-titer MOG-IgG were also found in patients with NMO (n = 3, 7%), HR-NMO (n = 7, 13%), CIS (n = 2, 6%), MS (n = 2, 3%) and CTRL (3, 3%) (Table 1, Figure 1). Median MOG-IgG titers of seropositive patients were 1:2,560 (1:160-1:2,560) in NMO, 1:2,560 (1:640-1:5,120) in HR-NMO, 1:2,560 (1:160-1:20,480) in ADEM, 1:640 and 1:5,120 in CIS, 1:160 and 1:160 in MS and 1:320 (1:160-1:640) in CTRL (Figure 1 and Table 1).

**Figure 2:**
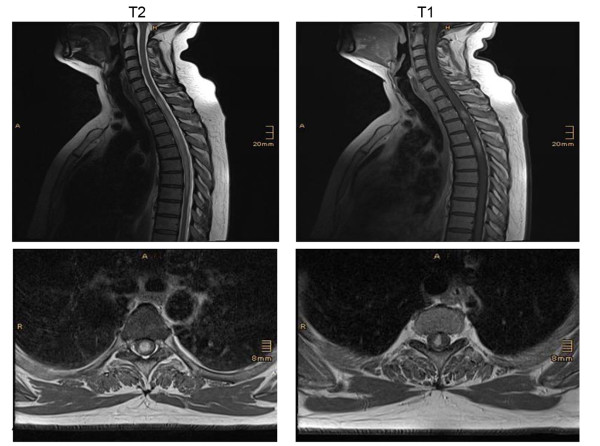
**Longitudinally extending spinal cord lesions in a high-titer MOG-IgG seropositive and AQP4-IgG seronegative patient**. Sagittal T2 weighted MRI of a lesion extending over 8 vertebral segments (upper left) in a 49 year old female patient (Table 2, patient number 10). Transversal T2 weighted image (lower left) shows the extension over the whole cross-sectional area of the spinal cord. Contrast enhanced T1 weighted images (sagittal, upper right and transversal, lower right) show scattered contrast enhancement in the lesion.

The clinical characteristics of MOG-IgG positive patients with NMO, HR-NMO and CIS are shown in Table 2. The MOG-IgG positive NMO patients consisted of two AQP4-IgG seronegative patients (a two year old female child and a 56 year old male), both with a MOG-IgG titer of 1:2,560, and one patient (a 39 year old woman) who was double positive for both, MOG-IgG (titer 1:160) and AQP4-IgG (titer 1:1,280). Within the HR-NMO group, seven of 21 (33%) AQP4-IgG negative patients were positive for high-titer MOG-IgG (Table 1 and 2). These seven patients included five patients with recurrent ON and two patients with monophasic LETM. The spinal magnetic resonance image (MRI) of a high-titer MOG-IgG positive patient presenting with LETM (patient number 10, Table 2) is shown in Figure 2. Both MOG-IgG seropositive CIS patients presented with monophasic ON and were negative for AQP4-IgG (Table 2).

**Table 2:** Clinical characteristics of high-titer MOG-IgG seropositive patients with NMO (n = 3), HR-NMO (n = 7) and CIS (n = 2)

No./Sex/Age (y)	Diagnosis	MOG-IgG titer	AQP4-IgG titer	relapses	ON	myelitis	LETM	Cerebral MRI lesions	MOG -TCC
1/F/2	NMO	1:2,560	negative	2	yes	yes	yes	no	pos

2/F/39	NMO	1:160	1:1,280	4	yes	yes	yes	no	n.a

3/M/56	NMO	1:2,560	negative	2	yes	yes	yes	n.a	pos

4/F/28	HR-NMO (rON)	1:5,120	negative	2	yes	no	no	no	pos

5/M/13	HR-NMO (rON)	1:5,120	negative	3	yes	no	no	no	pos

6/F/48	HR-NMO (rON)	1:2,560	negative	16	yes	no	no	no	pos

7/F/46	HR-NMO (rON)	1:640	negative	3	yes	no	no	no	neg

8/M/32*	HR-NMO (rON)	1:640	negative	11	yes	no	no	no	neg

9/F/43	HR-NMO (LETM)	1:2,560	negative	1	no	yes	yes	no	pos

10/F/49*	HR-NMO (LETM)	1:640	negative	1	no	yes	yes	no	pos

11/F/5	CIS (ON)	1:5,120	negative	1	yes	no	no	no	pos

12/M/13	CIS (ON)	1:640	negative	1	yes	no	no	no	neg

Abbreviations: No. = patient number, F = female, M = male, y = years, MRI = magnetic resonance image, NMO = Neuromyelitis optica, HR-NMO = High-risk NMO, rON = recurrent optic neuritis, ON = optic neuritis, LETM = longitudinally extensive transverse myelitis, CIS = clinically isolated syndrome, MOG-TCC = MOG-IgG dependent terminal complement complex, n.a = not available. * paired serum and CSF samples were available from patient 8 and 10. The corresponding CSF was found to be negative for AQP4-IgG as well as for MOG-IgG (CSF was analyzed undiluted and diluted 1:2 as previously described [48]).

Furthermore, MOG-IgG was detected at threshold levels (1:160) in two of 71 MS patients (secondary progressive MS and pediatric MS). Within the CTRL cohort, MOG-IgG was observed in two of 27 SLE patients (1:320 and 1:160) and one of 24 OND patients (1:640, pediatric patient with genetically confirmed citrullinemia, presenting with encephalopathy and multifocal neurological deficits [24]), whereas all 50 healthy controls were MOG-IgG negative.

### Analysis of AQP4-IgG and MOG-IgG mediated complement activation using MOG or AQP4 transfected HEK-293A cells

We additionally analyzed a subset of 15 AQP4-IgG positive samples for the presence of IgG1-IgG4 isotypes, and found that AQP4-IgG antibodies consisted primarily of the IgG1 isotype in 13 patients (87%), while two patients presented with IgG1 and IgG3 antibodies (13%). In contrast to anti-AQP4 autoantibodies, analysis of IgG1-IgG4 isotypes revealed that human serum MOG-IgG antibodies of 15 investigated patients consisted only of the IgG1 isotype.

**Figure 3:**
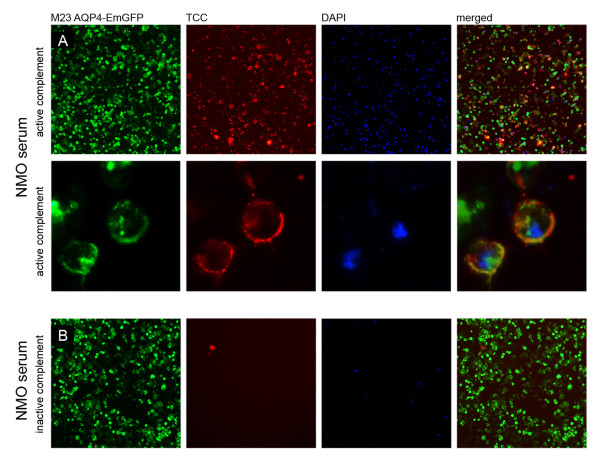
**Serum AQP4-IgG antibodies of an NMO patient activate the complement cascade in the presence of active complement resulting in the deposition of the terminal C5b-9 complement complex**. A. Formation of the terminal complement complex (TCC, red) on the surface of M23 AQP4-EmGFP expressing HEK-293A cells (green) following addition of a heat- inactivated AQP4-IgG positive serum sample (titer of 1:10,240). The membrane attack complex resulted in lysis of AQP4-expressing cells (DAPI, blue). B. No antibody mediated complement activation was detectable following incubation of the same heat-inactivated AQP4-IgG positive NMO serum sample supplemented with inactive complement (TCC, red). Images are representative of similar staining patterns observed in independent complement activation assays of the 27 AQP4-IgG positive patients (Table 3). Images are shown in 20 × (A-upper panel and B) and 63 × (A-lower panel) magnification.

**Figure 4:**
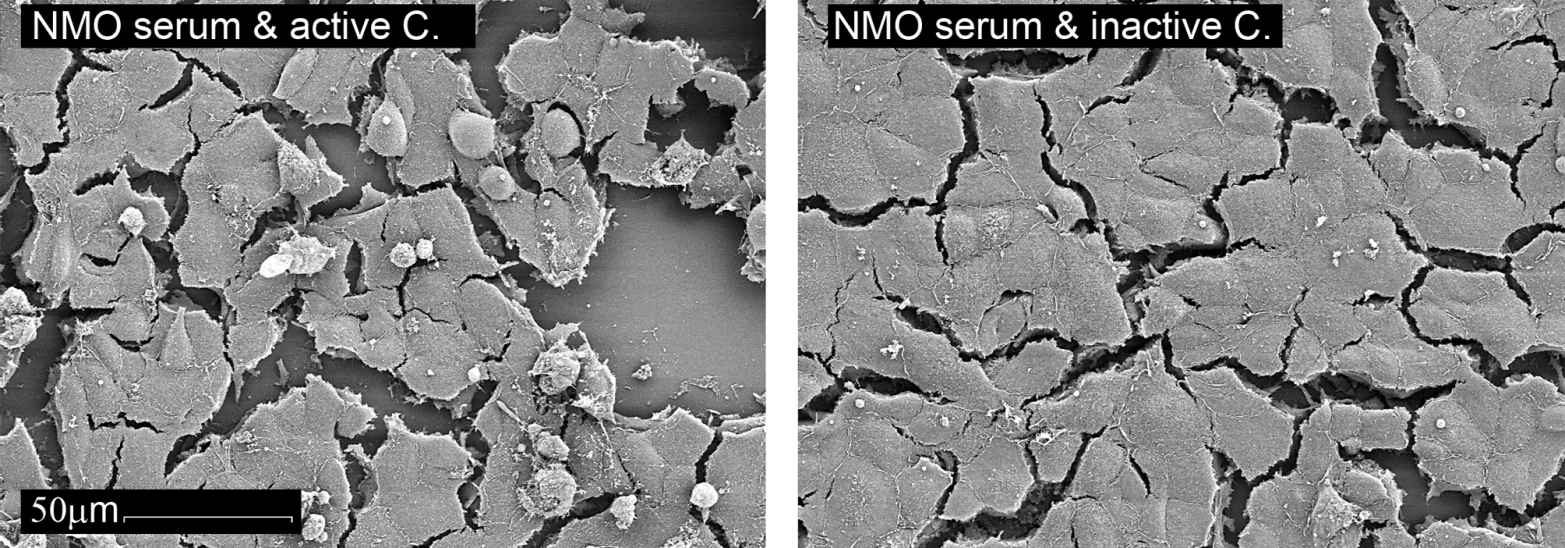
**Scanning electron microscopy of serum AQP4-IgG mediated complement activation**. Following incubation of AQP4 transfected HEK-293A cells with an NMO serum (AQP4-IgG titer: 1:10,240) and either active or inactive complement, the cells were investigated via scanning electron microscopy (1,000 × magnification). NMO serum supplemented with active complement resulted in increased apoptosis (shown by the detached cells, left panel). In contrast, the cell layer of NMO serum supplemented with inactive complement remained largely preserved (right panel). Images are representative of similar observations in independent complement activation assays.

**Figure 5:**
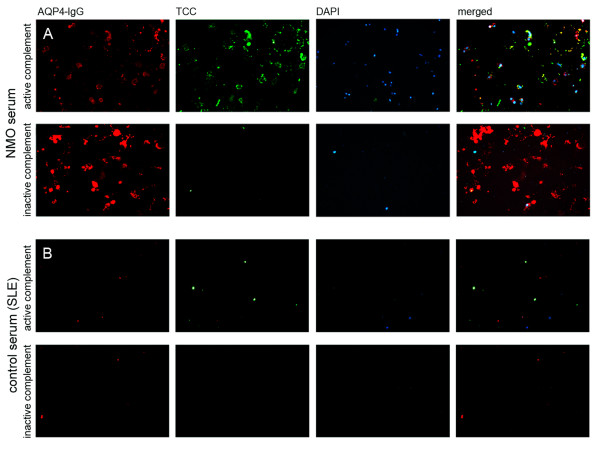
**Co-localization of the terminal complement complex (TCC) with an NMO patient's serum AQP4-IgG**. A. The heat-inactivated serum sample of an AQP4-IgG positive NMO patient (titer 1:5,120) was added to M23 AQP4 transfected cells (without EmGFP fusion protein) in the presence of either active or inactive complement. Complement-mediated cytotoxicity (TCC, green) was only detectable after addition of active complement. Furthermore, the TCC co-localized (merged) with the antibodies directed to AQP4 (AQP4-IgG, red) resulting in lysis of the AQP4 transfected cells (DAPI staining, blue). The activation of the complement cascade was accompanied by an internalization of AQP4-IgG, resulting in an attenuated signal (red). B. An AQP4-IgG negative serum sample of an SLE patient resulted in no formation of the TCC in the presence of active complement.

Using our live cell staining immunofluorescence assay (IF) assay, we found that human AQP4-IgG are able to activate the complement cascade at high-titers, leading to the formation of the terminal complement complex (TCC). The resultant TCC was exclusively detected on the surface of AQP4-EmGFP transfected cells (Figure 3). Furthermore, NMO antibody mediated complement activation resulted in complement-dependent lysis of AQP4 transfected cells, which could be demonstrated via DAPI staining of dead cells (Figure 3). Scanning electron microscopy analysis revealed increased apoptosis characterized by a detachment of the cell layer (Figure 4). No TCC formation was observed using AQP4-IgG positive serum samples supplemented with inactive complement. Incubation of AQP4 transfected cells with active complement without serum or with serum samples of AQP4-IgG negative patients supplemented with active complement did not result in complement dependent cytotoxicity (CDC; additional file 1). To verify the antibody mediated localization of the TCC, cells were transfected using AQP4 without the EmGFP fusion protein (Figure 5). In this setting, the membrane attack complex co-localized with human AQP4-IgG. Furthermore, complement-dependent internalization of AQP4-IgG antibodies was observed.

**Figure 6:**
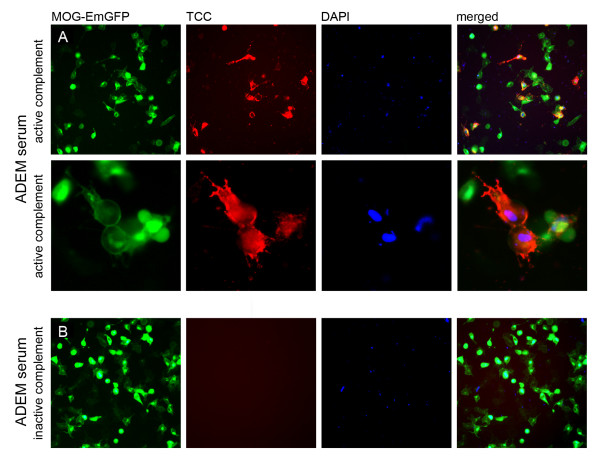
**Serum MOG-IgG mediated complement activation**. A. High-titer MOG-IgG antibodies of an ADEM patient (titer 1:20,480) can activate the complement system on MOG expressing HEK-293A cells (MOG-EmGFP, green), resulting in TCC formation (red) and cell lysis (blue) following addition of active complement. B. No TCC formation was observed with the MOG-IgG positive serum when incubated with inactive complement, resulting in fewer DAPI stained cells (blue). Images are representative of similar staining patterns observed in independent complement activation assays of the 17 MOG-IgG positive patients (Table 3). Images are shown in 20 × (A-upper panel and B) and 63 × (A-lower panel) magnification.

MOG-IgG antibodies were able to induce the complement cascade *in vitro *in the same manner as shown for AQP4-IgG antibodies. Using MOG transfected cells with and without EmGFP fusion protein, we could clearly show a co-localization of the TCC with MOG-EmGFP (Figure 6). The membrane attack complex resulted in an internalization of anti-MOG antibodies and complement-mediated lysis of MOG transfected cells (Figure 6, additional file 2). In order to exclude an unspecific activation of complement by MOG itself [31], MOG transfected cells were incubated with active complement in the absence of serum (additional file 2). In contrast to high-titer MOG-IgG positive serum samples of ADEM, NMO, HR-NMO and CIS patients, low-titer and MOG-IgG negative samples did not lead to CDC in the presence of active complement (additional file 2).

### AQP4-IgG and MOG-IgG directed complement-mediated cytotoxicity in patients with CNS demyelinating diseases

Next, we grouped patients with NMO (n = 23), HR-NMO (n = 33), ADEM (n = 19), CIS (n = 14), MS (n = 10) and CTRL (n = 14) based on their ability to initiate MOG-IgG or AQP4-IgG dependent complement activation (TCC AQP4-/MOG-, TCC AQP4+/MOG- or TCC AQP4-/MOG+; Table 3 and additional file 3). The selection of patients for the analysis of complement-mediated cytotoxicity was based on the availability of serum samples and the use of samples which are representative for our entire study population. We found no significant differences in the three groups regarding clinical parameters, such as ON, myelitis, LETM, disease duration or relapse frequency (data not shown).

**Table 3:** Patients with CNS demyelinating diseases and controls according to their serum AQP4-IgG and MOG-IgG mediated complement activation measured by the formation of the terminal complement complex (TCC)

	TCC AQP4-MOG-	TCC AQP4+MOG-	TCC AQP4-MOG+	p-value
Number	69	27	17	

Females^1^	44 (64%)	25 (93%) *§	11 (65%)	0.017

Disease^2^				
NMO (n = 23)	6 (26%)	15 (65%)	2 (9%)	< 0.001
HR-NMO (n = 33)	17 (52%)	11 (33%)	5 (15%)	
ADEM (n = 19)	11 (58%)	0 (0%)	8 (42%)	
CIS (n = 14)	12 (86%)	1 (7%)	1 (7%)	
MS (n = 10)	10 (100%)	0 (0%)	0 (0%)	
CTRL (n = 14)	13 (93%)	0 (0%)	1 (7%)	

Age (y)^3^	38 (8-74)	48 (19-80) *	13 (2-56) *#	< 0.001

AQP4 IgG^1^	16 (23%)	27 (100%) *§	0 (0%) *	< 0.001
Titer (1:)^3^	0	1,280 *§	0	< 0.001
	(0-1,280)	(160-20,480)	(0)	

MOG IgG^1^	7 (10%)	0 (0%)	17 (100%) *#	< 0.001
Titer (1:)^3^	0	0	2,560 *#	< 0.001
	(0-2,560)	(0-20)	(640-20,480)	

Patients with NMO (n = 23), HR-NMO (n = 33), ADEM (n = 19), CIS (n = 14), MS (n = 10) and CTRL (n = 14) were grouped according to TCC formation: TCC AQP4 and MOG negative patients (AQP4-MOG-), TCC AQP4 positive and MOG negative (AQP4+MOG-) and TCC AQP4 negative and MOG positive (AQP4-MOG+). In detail, the antibody serostatus (AQP4-IgG/MOG-IgG) and titer levels of the patients investigated for TCC formation are listed in the additional file 3. P-value: groups were compared using^1 ^Fisher's exact test,^2 ^Chi-square test and^3 ^Mann-Whitney U test (data are shown as median with range); * statistically different from TCC AQP4-MOG-; # statistically different from TCC AQP4+MOG-; § statistically different from TCC AQP4-MOG+ (p-values were corrected for multiple comparisons). Abbreviations: TCC = terminal complement complex, y = years.

Overall, AQP4-IgG mediated complement activation was observed exclusively in 27 patients positive for AQP4-IgG with a median titer of 1:1,280 (ranging from 1:160 to 1:20,480; Table 3 and additional file 3), consisting of NMO, HR-NMO and one CIS patient. In contrast, samples of patients with lower AQP4-IgG antibody titers and all investigated serum samples of AQP4-IgG negative CNS demyelinating diseases and controls were not able to activate the complement cascade on the surface of AQP4-expressing HEK-293A cells (Table 3 and additional file 3).

Similarly, assembly of the TCC was observed on the surface of MOG transfected cells after incubation with serum samples having a median MOG-IgG titer level of 1:2,560 (ranging from 1:640 to 1:20,480), as shown in Table 3 and additional file 3. Within the group of subjects investigated for CDC, MOG-IgG dependent TCC formation was found in 2/23 (9%) definite NMO, 5/33 (15%) HR-NMO, 8/19 (42%) ADEM, 1/14 (7%) CIS and 1/14 (7%) CTRL (pediatric patient with genetically confirmed citrullinemia with a MOG-IgG serum titer of 1:640). MOG-IgG mediated complement activation did not correlate with clinical parameters (data not shown), but was associated with a younger age of the investigated patients (Table 3). In contrast, subjects with lower MOG-IgG titers and all MOG-IgG negative patients (NMO, HR-NMO, ADEM, CIS, MS and CTRL) did not activate the complement cascade on the surface of MOG transfected cells.

## Discussion

In this multicenter study we describe for the first time the presence of serum high-titer MOG-IgG antibodies in patients with NMO and HR-NMO. Our data confirm several studies demonstrating the presence of MOG-IgG in a subgroup of patients with ADEM [24-29], as well as AQP4-IgG in NMO and HR-NMO [1,2,9,10,12,14,15,32]. Moreover, we report the occurrence of MOG-IgG antibodies in AQP4-IgG seronegative patients with either NMO (two of two) or HR-NMO (seven of 21), and in monophasic ON/CIS patients (two of 32). These results suggest that MOG is a target antigen in AQP4-IgG negative patients with NMO and HR-NMO, which to our knowledge has not been described before. This is of particular relevance since AQP4-IgG is absent in approximately 5-40% of these patients. This variability of AQP4 antibody detection could depend on the antibody assay, the AQP4 isoform, as well as the study population and/or prior immunosuppressive treatment [9,12,14-16].

However, it has been speculated whether different pathomechanisms are involved in AQP4-IgG seronegative NMO and HR-NMO patients compared to subjects with "AQP4 autoimmune channelopathies". This assumption is supported by findings showing no development of NMO-like symptoms in animals immunized with purified antibodies from AQP4-IgG seronegative NMO patients [33]. In contrast, antibodies from AQP4-IgG positive NMO patients were shown to be pathogenic after intra-cerebral administration combined with human complement [34], as well as following EAE induction [33,35,36].

An involvement of antibodies directed against MOG in NMO and HR-NMO is encouraged by *in vivo *studies demonstrating the spontaneous development of human NMO-like symptoms in a double-transgenic mouse strain with opticospinal EAE [19,20]. Expressing T cell and B cell receptors specific for MOG, these mice showed inflammatory demyelinating lesions in the optic nerve and spinal cord, sparing brain and cerebellum [19]. In addition, the animals harbored a MOG-IgG1 directed humoral immune response [19]. Several studies demonstrated the induction of an NMO-like disease in distinct rat strains following immunization with MOG [21-23]. However, at present only limited information is available regarding MOG-IgG antibodies in human patients suffering from NMO or HR-NMO symptoms and related disorders. One study revealed the presence of antibodies directed to the bacterially produced extracellular domain of recombinant MOG, as investigated with ELISA and immunoblot, in four NMO patients [30]. However, it was shown that the detection of antibodies against natively folded MOG is restricted to assays using MOG expressed on the surface of cells. In contrast, commonly applied ELISA or Western blot assays using the bacterially expressed protein failed to identify these antibodies. This might provide an explanation for the controversial results regarding serum MOG-IgG antibodies in MS patients. Furthermore, high-titer MOG-IgG was detected in a subgroup of patients with pediatric ADEM and MS, but only rarely in adult-onset MS [24-27,37]. However, these studies did not include patients with definite and HR-NMO. Most recently, we could demonstrate high-titer MOG-IgG antibodies in pediatric patients with recurrent ON (Rostasy K, Mader S, Schanda K, Huppke P, Gärtner J, Kraus V, Karenfort M, Tibussek D, Blaschek A, Kornek B, Leitz S, Schimmel M, Di Pauli F, Berger T, Reindl M: Anti-MOG antibodies in children with optic neuritis, in press). Now we describe the presence of MOG-IgG in NMO and HR-NMO. These findings expand the heterogenous spectrum of MOG-IgG mediated human demyelinating diseases from ADEM and pediatric MS to now include AQP4-IgG seronegative recurrent ON, LETM and NMO. Nevertheless, MOG might not be the only autoantigen present in AQP4-IgG seronegative patients with NMO and related disorders. Recent studies have described antibodies to NMDA-type glutamate receptors or CV2/CRMP5 in AQP4-IgG seronegative cases with NMO or ON [38-40]. Therefore, our findings of MOG-IgG support a possible relevance of several specific CNS autoantigens in AQP4-IgG seronegative NMO and HR-NMO cases. Further studies are now required in order to identify potential target antigens.

Several *in vitro *studies have demonstrated the pathogenic effect of AQP4-IgG in the presence of active complement [32,41,42], which is confirmed by our findings of AQP4-IgG1 mediated CDC at high-titer serum levels of AQP4-IgG. Furthermore, our results show that antibodies against MOG were primarily of the IgG1 subtype and could activate the complement cascade *in vitro*, resulting in the formation of the TCC on living MOG transfected HEK-293A cells. To our knowledge, these observations are novel and might provide a deeper insight into the role of high-titer serum anti-MOG antibodies. Thus, the detection of high-titer MOG-IgG might not only serve as a valuable biomarker in AQP4-IgG negative NMO and HR-NMO patients, but possibly play a role as pathogenic factor in human demyelinating diseases, although this needs to be further investigated.

There are two limitations that need to be addressed regarding our study. The first limitation concerns the usage of an immunofluorescence assay to measure AQP4-IgG and MOG-IgG antibodies and TCC formation. This is often criticised by other researchers using automated assays like flow cytometry or immunoprecipitation for the measurement of specific antibodies. However, experiences from the last decades have strongly emphasized that immunofluorescence assays are the gold standard for the detection of several autoantibodies, such as anti-nuclear antibodies. Furthermore, immunofluorescence assays were shown to yield the highest sensitivity for the detection of AQP4-IgG [9,14,43-45]. In addition, as an important quality control in our study, all samples were evaluated by three independent investigators with 100% concordance rate. The second limitation concerns the low number of AQP4-IgG seronegative NMO patients in our study population. Nevertheless, we believe that our findings are of high importance as a substantial proportion of NMO and HR-NMO patients lack a specific biomarker. Hence, our results need to be confirmed in a larger study cohort of AQP4-IgG negative NMO and HR-NMO subjects.

## Conclusions

We could show for the first time that AQP4-IgG antibody seronegative patients with NMO and HR-NMO harbor a MOG-IgG directed immune response. MOG is not a target antigen in "AQP4 channelopathies", raising the question of whether MOG-IgG positive NMO and HR-NMO patients share a possible disease overlap with MOG-IgG positive ADEM. Overall, these results are highly relevant for clinical practice in order to optimize patients' treatment, and might help to elucidate the disease pathomechanisms of these rare CNS demyelinating diseases.

## Methods

### Patients and serum samples

The following patients were recruited from Austria (n = 295), Germany (n = 19), Slovakia (n = 2) and Serbia (n = 19) (Table 1): (1) a total of 45 NMO patients diagnosed according to the revised diagnostic criteria from Wingerchuk et al., 2006 [3], (2) 53 patients with a high risk of developing NMO (HR-NMO) including 28 monophasic LETM, 13 recurrent LETM and 12 recurrent ON subjects [3,10], (3) 33 patients fulfilling the diagnostic criteria for ADEM [46], (4) 32 CIS patients comprising 19 myelitis (59%) and 13 ON (41%), (5) 71 patients with MS according to the revised "McDonald Criteria" 2005 [47] including 44 patients with relapsing remitting MS, 8 patients with primary progressive MS and 19 patients with secondary progressive MS, (6) 101 controls including 24 patients with OND (stroke, Parkinson's disease, epileptic seizure, radiculopathy, insomnia, sleep apnoea syndrome, CNS lymphoma, traumatic brain injury, myasthenia gravis, chronic inflammatory demyelinating polyneuropathy, vestibular neuritis, orthostatic syncope, psychogenic neurological symptoms, CNS vasculitis, hereditary neuropathy, analgesic-induced headache, neuroborreliosis, viral encephalitis, chronic tension-type headache, glioblastoma multiforme), 27 patients with SLE and 50 healthy blood donors obtained from the central institute for blood transfusion (Central Institute for Blood Transfusion and Immunological Department, Innsbruck University Hospital).

External serum samples were shipped on dry ice to Innsbruck and all samples were stored at -20°C until analysis. The present study was approved by the ethical committee of Innsbruck Medical University (study no. UN3041 257/4.8, 21.09.2007) and all Austrian patients or parents/legal guardians gave written informed consent to the study protocol. All Serbian and Slovakian patients gave their informed consent for serum sampling and this study was approved by the Institutional Review Board of the Clinic of Neurology, Clinical Center of Serbia, Belgrade. The Slovakian patients signed the translated informed consent form of the Innsbruck Medical University. All German samples were tested anonymously as requested by the institutional review board of the University of Heidelberg.

### AQP4-IgG and MOG-IgG immunofluorescence assays

Analysis of M23 AQP4-IgG was performed using a live cell staining IF assay as recently described [9,33,48].

HEK-293A cells (ATCC, LGC Standards GmbH, Wesel, Germany) were transiently transfected (Fugene 6 transfection reagent, Roche, Mannheim, Germany) using the Vivid Colours™ pcDNA™ 6.2C-EmGFP-GW/TOPO plasmid (Invitrogen, Carlsbad, CA, USA), expressing M23 AQP4 fused C-terminally to an emerald green fluorescence protein (EmGFP). The AQP4-IgG IF assay was performed by blocking the transfected cells with 4 μg/ml goat IgG (Sigma-Aldrich, St. Louis, MO, USA) diluted in PBS/10% FCS (Sigma-Aldrich), subsequently incubating the cells with pre-absorbed serum samples (rabbit liver powder, Sigma-Aldrich) at a 1:20 and 1:40 dilution for one hour at 4°C. Bound antibodies were detected using Cy™3-conjugated goat anti-human IgG antibody (Jackson ImmunoResearch Laboratory, West Grove, PA, USA) for 30 minutes at room temperature. Dead cells were excluded by DAPI staining (Sigma-Aldrich). The AQP4-IgG status was determined using a fluorescence microscope (Leica DMI 4000B, Wetzlar, Germany). Each serum sample was individually evaluated by three independent, clinically blinded investigators (SM, KS and VG), yielding a concordance rate of 100%.

In order to determine AQP4-IgG titer levels, AQP4-IgG seropositive samples were further diluted until loss of specific antibody staining. AQP4-IgG was purified from a NMO patient's plasma exchange material as recently described [33] and served as positive control for each assay.

Serum MOG-IgG was determined in pre-absorbed samples using HEK-293A cells transiently transfected with human MOG cloned into the mammalian expression vector Vivid Colours™ pcDNA™ 6.2 C-EmGFP/TOPO (Invitrogen), expressing MOG fused C-terminally to EmGFP as previously reported [24]. Serum MOG-IgG was detected on the surface of MOG expressing cells, using Cy™3-conjugated goat anti-human IgG antibody. Titer levels were determined for MOG-IgG positive samples by serial dilution of serum until loss of signal. Based on our previous results, the cut-off value of high-titer MOG-IgG antibodies was defined as ≥ 1:160, with 100% specificity compared to healthy controls [24]. Consequently, in this study patients with titers ≥ 1:160 are defined as being "high-titer positive". In contrast, patients with "low serum antibody reactivity" (1:20-1:80) as well as serum antibody negative samples (with no detectable antibodies, titer = 0) were summarized as "negative" cohort, to simplify the data sets. However, titer levels below 1:160 are mentioned whenever relevant and illustrated in Figure 1. Additionally, antibodies purified from the plasma exchange material of a high-titer MOG-IgG positive ADEM patient were added as a quality control to each assay. Dead cells were excluded by DAPI staining and the presence and titer levels of MOG-IgG were analyzed by three clinically blinded and experienced investigators (SM, KS and VG).

In order to exclude unspecific background staining, we additionally performed serum antibody stainings using untransfected HEK-293A cells for both IF assays, transfected cells expressing the fusion protein (EmGFP) as a control for the AQP4-IgG assay as well as CD2-EmGFP transfected cells (another protein of the immunoglobulin superfamily) for the MOG-IgG assay. Non-specific background binding was clearly distinguishable from a specific antibody staining in our immunofluorescence setting.

Furthermore, MOG-IgG and AQP4-IgG seropositive and seronegative control samples are regularly retested for antibody titer levels to ensure the quality of the testing system. Titer levels remain constant in the serum samples which are stored at -20°C, even two years after first analysis.

### Determination of IgG1-IgG4 isotypes

Serum antibodies to MOG and AQP4 were analyzed in a subgroup of 15 patients for IgG1-IgG4 isotypes via our live cell staining IF assay using MOG or AQP4 transfected cells. After blocking with goat IgG, the transfected cells were incubated with the pre-absorbed serum samples (1:20 and 1:40 dilution) for one hour. Subsequently, cells were washed and incubated with mouse monoclonal anti-human IgG1-IgG4 isotype antibodies for 30 minutes (Sigma-Aldrich, 1:100 dilution in PBS/10% FCS), followed by detection using Alexa Fluor^® ^546 goat anti-mouse IgG (Invitrogen) for 30 minutes. Dead cells were excluded by DAPI staining and analysis was performed by three independent investigators (SM, KS and VG).

### Antibody mediated terminal complement complex (TCC) in cells expressing AQP4 or MOG

Antibody mediated complement activation was investigated in 23 NMO, 33 HR-NMO, 19 ADEM, 14 CIS, 10 MS and 14 CTRL. The selection of patients for the analysis of complement-mediated cytotoxicity was based on the availability of serum samples and the use of samples which are representative for our entire study population. Briefly, serum samples and human complement (Sigma-Aldrich) were heat-inactivated at 56°C for 45 minutes. Inactivated serum samples were diluted 1:10 in serum-free X-VIVO 15 medium (Lonza, Verviers, Belgium) and pre-absorbed with rabbit liver powder. Cells expressing either MOG or AQP4 were washed three times with X-VIVO 15 medium and subsequently incubated with heat-inactivated, pre-absorbed serum samples and 20% active versus 20% heat-inactivated human complement for 90 minutes at 37°C. After washing the cells three times with 100 μl X-VIVO 15 medium, detection of TCC formation was performed by adding the murine-monoclonal anti-human SC5b-9 (Quidel, San Diego, CA, USA; diluted 1:200 in X-VIVO 15) for one hour at 4°C. Following a 30 minutes incubation with the fluorescence labelled Alexa Fluor^® ^546 goat anti-mouse IgG antibody (1:300, Invitrogen), cells were washed with PBS/10% FCS and dead cells were visualized by DAPI staining. All samples were assessed for the presence of the surface membrane attack complex by three independent investigators blinded for clinical information as well as the design of the assay concerning usage of active/inactive complement (SM, KS and VG).

To analyze the co-localization of the TCC and serum MOG-IgG or AQP4-IgG, we used HEK-293A cells expressing MOG or AQP4 without EmGFP fusion protein.

To obtain M23 AQP4 without EmGFP fusion protein, the M23 AQP4 isoform was cloned into the pcDNA3.1 Directional TOPO Expression vector (Invitrogen) [9]. In order to conduct experiments with transfected cells expressing MOG without EmGFP fusion protein, we cloned MOG into the pCMV vector (Invitrogen).

Briefly, after incubating the cells with heat-inactivated samples and active versus inactive complement (90 minutes, 37°C), the cells were washed (X-VIVO 15) and stained with the murine-monoclonal anti-human SC5b-9 (Quidel, diluted 1:200 in X-VIVO 15 medium, one hour, room temperature) as described above. Following three washing steps, the Alexa Fluor^® ^488 goat anti-mouse IgG antibody and Cy™3-conjugated goat anti-human IgG antibody were diluted in X-VIVO 15 medium and incubated for 30 minutes. Co-staining of AQP4-IgG and MOG-IgG antibodies (red) and TCC (green) was investigated in a blinded fashion (SM, KS and VG), and dead cells were visualized by DAPI staining. Control experiments using active complement in the absence of serum showed no TCC formation on MOG or AQP4 expressing cells (additional file 1 and additional file 2). Additionally, no cross reaction of the antibodies to other species than stated was observed.

### Scanning electron microscopy (SEM)

AQP4-IgG mediated complement activation was confirmed via SEM. Briefly, HEK-293A cells were seeded on poly-L-lysine (Sigma-Aldrich) coated glass slides (Menzel, Braunschweig, Germany) and transiently transfected with the AQP4-EmGFP vector. Thereafter, serum samples supplemented with either active or inactive complement were added to the cells. Following incubation at 37°C for 90 minutes, the cells were washed with PBS and fixed in glutaraldehyde (2.5%, v/v in 0.1 m PBS, pH 7.4). After incubation for 30 minutes at room temperature, the fixative was replaced by fresh fixative, and incubated for another two hours at room temperature. Subsequently, complement activation was investigated via SEM according to standard procedures [49]. Samples were viewed for complement activation in a blinded fashion using the field-emission SEM DSM982-Gemini (ZEISS, Oberkochen, Germany).

### Statistical analysis

Statistical analysis (means, medians, range, standard deviations) and significance of group differences were done using IBM SPSS software (release 18.0, SPSS Inc., USA) or GraphPad Prism 5 (GraphPad, San Diego, USA). Between-group comparisons were performed with Kruskal-Wallis test, Dunn's multiple comparison post-hoc test, Mann-Whitney *U *test, Fisher's exact test and Chi-square test as appropriate. Correlation of parameters was analyzed with Spearman's non-parametric correlation. Statistical significance was defined as two-sided p-value less than 0.05 and Bonferroni's correction was applied for multiple comparisons when appropriate.

## Competing interests

The authors declare that they have no competing interests.

## Authors' contributions

SM, VG, KS and MR conceived and designed the experiments. SM, VG and KS carried out all experiments. SM and MR analysed and interpreted the data. KP performed the scanning electron microscopy. KR, ID, AL, SJ, FDP, BK, RE, FD, FAE, MS, PK, JD, WK, TB and MR participated in serum and data collection. SM, VG, KS and MR wrote the initial manuscript. All authors have read and approved the final version of the manuscript.

## Supplementary Material

Additional file 1 **Complement dependent cytotoxicity on the surface of AQP4 transfected cells occurs exclusively in AQP4-IgG positive serum samples**. Heat incativated serum samples of patients with NMO (AQP4-IgG positive), LETM (MOG-IgG positive) and ADEM (MOG-IgG positive and negative) were incubated on AQP4-EmGFP (green) expressing cells in the presence of active complement, and were analysed for AQP4-IgG mediated complement activation (TCC, red). The serum of an AQP4-IgG positive NMO patient together with active complement resulted in TCC formation, and an increased number of dead cells (blue, DAPI staining). Additionally, we observed a co-localization of the TCC (red) with the AQP4-EmGFP transfected cells (green), which is shown in the merged picture of the NMO patient. In contrast to the NMO patient, AQP4-IgG negative serum samples of patients with LETM or ADEM did not result in TCC formation in the presence of active complement. As an additional control, active complement without serum samples was added, showing no AQP4-IgG mediated complement activation. Click here for file

Additional file 2 **Complement dependent cytotoxicity on the surface of MOG transfected cells is restricted to the presence of serum high-titer MOG-IgG**. Heat inactivated serum samples of patients with NMO (AQP4-IgG positive), LETM (MOG-IgG positive), ADEM (MOG-IgG positive and negative) were incubated on MOG-EmGFP (green) transfected cells supplemented with human active complement. MOG-IgG specific complement activation (TCC, red) was observed using high-titer MOG-IgG positive sera of a patients with LETM and ADEM. Furthermore, the TCC co-localized with the MOG-EmGFP transfected cells (merged), resulting in an increased number of dead cells (blue, DAPI staining). Serum MOG-IgG negative patients (NMO and ADEM), as well as active complement (without serum) did not result in TCC formation. Click here for file

Additional file 3 **AQP4-IgG and MOG-IgG serostatus of the patients (Table 3) investigated for antibody mediated complement activation**. AQP4-IgG or MOG-IgG TCC formation in patients with NMO, HR-NMO, ADEM, CIS, MS and CTRL, which were subdivided according to their antibody serostatus: AQP4-IgG positive and MOG-IgG negative (AQP4+MOG-), AQP4-IgG negative and MOG-IgG seropositive (AQP4-MOG+) or double negative for AQP4-IgG and MOG-IgG (AQP4-MOG-). * = The AQP4-MOG- as well as AQP4+MOG- cohort includes patients with MOG-IgG titer levels below the threshold of 1:160 (cut-off), which are defined in our study population as MOG-IgG negative. Therefore, MOG-IgG titer levels below the threshold level are indicated as MOG titer (1:) *. Antibody titer levels are shown as median titer level (range). Abbreviation: TCC = terminal complement complex. Click here for file
