# Supplementary figures and images for: Complement activating antibodies to myelin oligodendrocyte glycoprotein in neuromyelitis optica and related disorders
Source: J Neuroinflammation. 2011 Dec 28;8:184. doi: 10.1186/1742-2094-8-184 (PMC3278385; doi:10.1186/1742-2094-8-184)

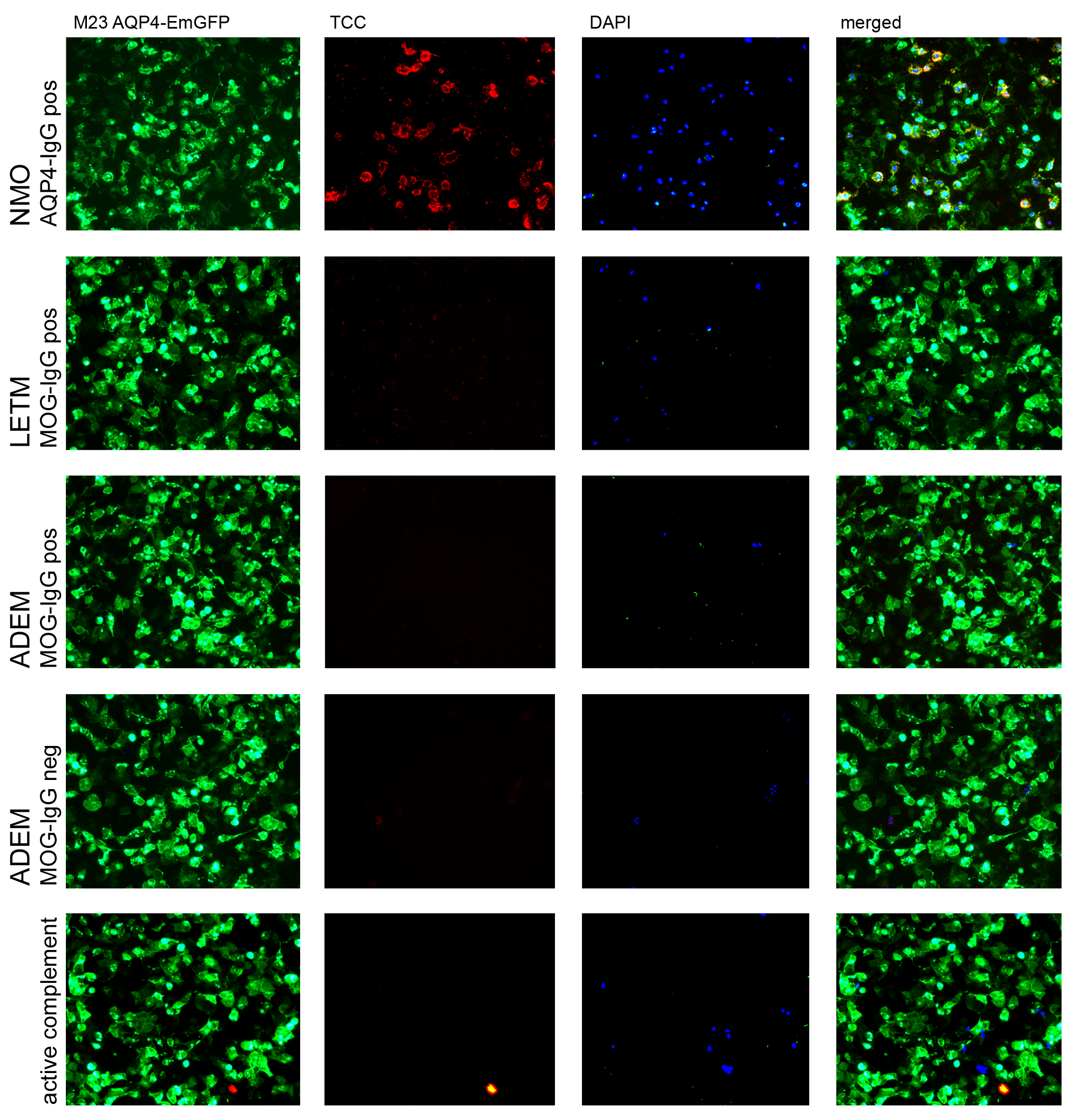

Supplement: Additional file 1 — Complement dependent cytotoxicity on the surface of AQP4 transfected cells occurs exclusively in AQP4-IgG positive serum samples. Heat incativated serum samples of patients with NMO (AQP4-IgG positive), LETM (MOG-IgG positive) and ADEM (MOG-IgG positive and negative) were incubated on AQP4-EmGFP (green) expressing cells in the presence of active complement, and were analysed for AQP4-IgG mediated complement activation (TCC, red). The serum of an AQP4-IgG positive NMO patient together with active complement resulted in TCC formation, and an increased number of dead cells (blue, DAPI staining). Additionally, we observed a co-localization of the TCC (red) with the AQP4-EmGFP transfected cells (green), which is shown in the merged picture of the NMO patient. In contrast to the NMO patient, AQP4-IgG negative serum samples of patients with LETM or ADEM did not result in TCC formation in the presence of active complement. As an additional control, active complement without serum samples was added, showing no AQP4-IgG mediated complement activation. [file 1742-2094-8-184-S1.TIFF]

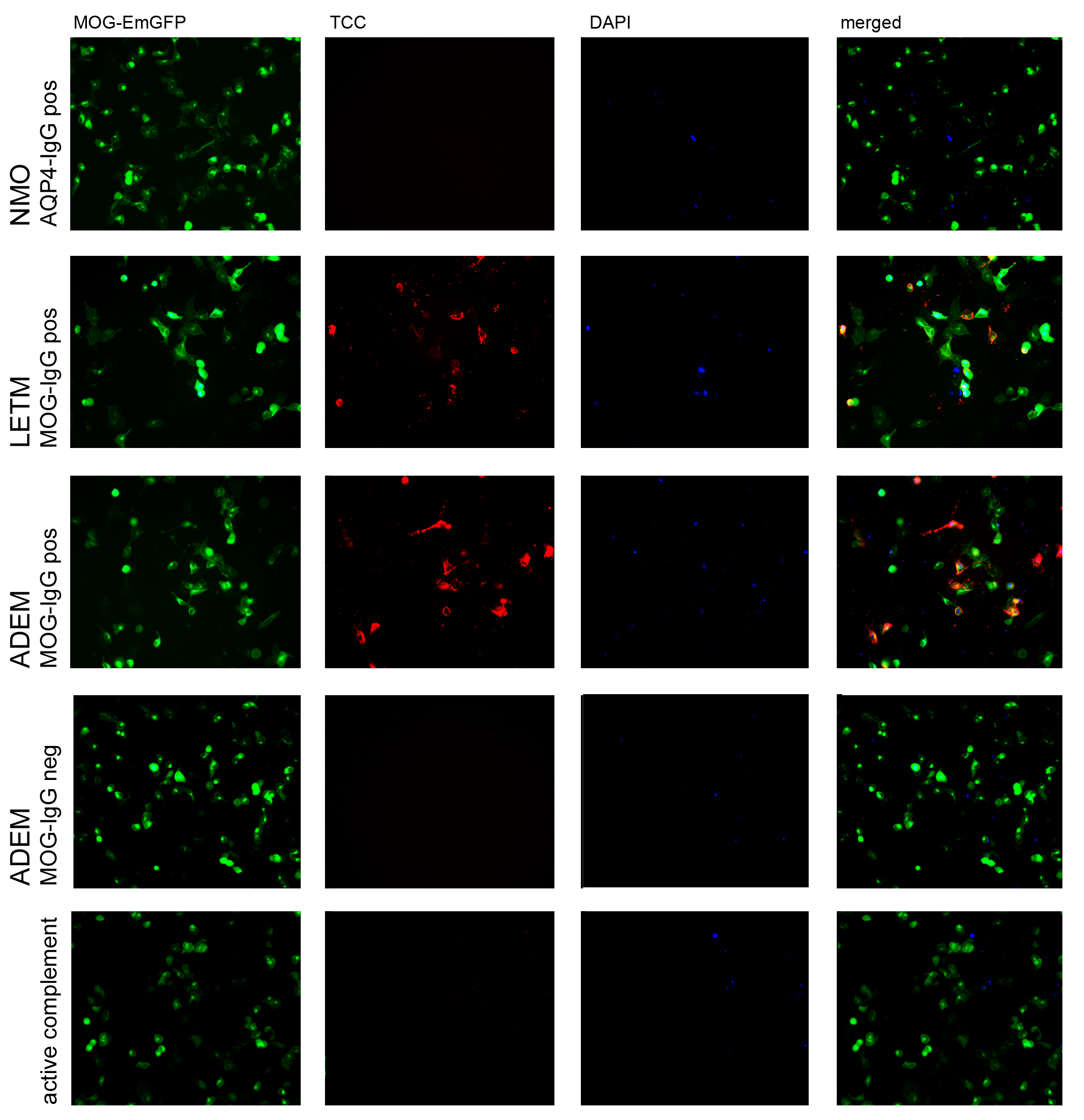

Supplement: Additional file 2 — Complement dependent cytotoxicity on the surface of MOG transfected cells is restricted to the presence of serum high-titer MOG-IgG. Heat inactivated serum samples of patients with NMO (AQP4-IgG positive), LETM (MOG-IgG positive), ADEM (MOG-IgG positive and negative) were incubated on MOG-EmGFP (green) transfected cells supplemented with human active complement. MOG-IgG specific complement activation (TCC, red) was observed using high-titer MOG-IgG positive sera of a patients with LETM and ADEM. Furthermore, the TCC co-localized with the MOG-EmGFP transfected cells (merged), resulting in an increased number of dead cells (blue, DAPI staining). Serum MOG-IgG negative patients (NMO and ADEM), as well as active complement (without serum) did not result in TCC formation. [file 1742-2094-8-184-S2.TIFF]
