# Supplementary material for: Complement activating antibodies to myelin oligodendrocyte glycoprotein in neuromyelitis optica and related disorders
Source: J Neuroinflammation. 2011 Dec 28;8:184. doi: 10.1186/1742-2094-8-184 (PMC3278385; doi:10.1186/1742-2094-8-184)
Supplement: Additional file 3 — AQP4-IgG and MOG-IgG serostatus of the patients (Table 3) investigated for antibody mediated complement activation. AQP4-IgG or MOG-IgG TCC formation in patients with NMO, HR-NMO, ADEM, CIS, MS and CTRL, which were subdivided according to their antibody serostatus: AQP4-IgG positive and MOG-IgG negative (AQP4+MOG-), AQP4-IgG negative and MOG-IgG seropositive (AQP4-MOG+) or double negative for AQP4-IgG and MOG-IgG (AQP4-MOG-). * = The AQP4-MOG- as well as AQP4+MOG- cohort includes patients with MOG-IgG titer levels below the threshold of 1:160 (cut-off), which are defined in our study population as MOG-IgG negative. Therefore, MOG-IgG titer levels below the threshold level are indicated as MOG titer (1:) *. Antibody titer levels are shown as median titer level (range). Abbreviation: TCC = terminal complement complex. [file 1742-2094-8-184-S3.DOC]

|  | **TCC**  **AQP4-MOG-** | **TCC**  **AQP4+MOG-** | **TCC**  **AQP4-MOG+** |
| --- | --- | --- | --- |
| **NMO (n=23)**  AQP4+MOG- (n=21)  AQP4 titer (1:)  MOG titer (1:) *  AQP4-MOG+ (n=2)  MOG titer (1:) | 6 (26%)  6  640 (320-640)  0 (0-20)  0  - | 15 (65%)  15  2,560 (160-20,480)  0 (0-20)  0  - | 2 (9%)  0  -  -  2  (2,560; 2,560) |
| **HR-NMO (n=33)**  AQP4+MOG- (n=21)  AQP4 titer (1:)  MOG titer (1:) *  AQP4-MOG+ (n=7)  MOG titer (1:)  AQP4-MOG-(n=5)  MOG titer (1:) * | 17 (52%)  10  320 (40-1,280)  0 (0-20)  2  (640; 640)  5  (0) | 11 (33%)  11  1,280 (640-10,240)  0 (0-20)  0  -  0  - | 5 (15%)  0  -  -  5  2,560 (640-5,120)  0  - |
| **ADEM (n=19)**  AQP4-MOG+ (n=12)  MOG titer (1:)  AQP4-MOG- (n=7)  MOG titer (1:) * | 11 (58%)  4  320 (160-2,560)  7  (0) | 0 (0%)  0  -  0  - | 8 (42%)  8  2,560 (640-20,480)  0  - |
| **CIS (n=14)**  AQP4+MOG- (n=1)  AQP4 titer (1:)  MOG titer (1:) *  AQP4-MOG+ (n=2)  MOG titer (1:)  AQP4-MOG- (n=11)  MOG titer (1:) * | 12 (86%)  0  -  -  1  (640)  11  0 (0-80) | 1 (7%)  1  (640)  (0)  0  -  0  - | 1 (7%)  0  -  -  1  (5,120)  0  - |
| **MS (n=10)**  AQP4-MOG- (n=10)  MOG titer (1:) * | 10 (100%)  10  0 (0-80) | 0 (0%)  0  - | 0 (0%)  0  - |
| **CTRL (n=14)**  AQP4-MOG+ (n=1)  MOG titer (1:)  AQP4-MOG- (n=13)  MOG titer (1:) * | 13 (93%)  0  -  13  0 (0-40) | 0 (0%)  0  -  0  - | 1 (7%)  1  (640)  0  - |
